# Sex and Aggression Characteristics in a Cohort of Patients with Pediatric Acute-Onset Neuropsychiatric Syndrome

**DOI:** 10.1089/cap.2021.0084

**Published:** 2022-10-17

**Authors:** Jaynelle Gao, Avis Chan, Theresa Willett, Bahare Farhadian, Melissa Silverman, Paula Tran, Sana Ahmed, Margo Thienemann, Jennifer Frankovich

**Affiliations:** ^1^ Department of Pediatrics, Stanford University of Medicine, Stanford, California, USA.; ^2^ Department of Psychiatry and Behavioral Sciences, Stanford University School of Medicine, Stanford, California, USA.

**Keywords:** PANS, PANDAS, aggression, MOAS, sex differences

## Abstract

**Objective:**

This study describes for the first time the characteristics by sex of patients with Pediatric Acute-onset Neuropsychiatric Syndrome (PANS), including clinical phenotype, treatment, and psychosocial aspects of disease.

**Methods:**

This cross-sectional study included 205 consecutive community patients evaluated between January 1, 2012 and March 30, 2019 and compared 87 females with 118 males. Our primary hypothesis was that males would display more aggression, as measured by the Modified Overt Aggression Scale (MOAS) and would be treated with immunotherapy earlier than females. The MOAS began to be administered 5 years into the study period, and 57 of the 205 families completed the MOAS for this study.

**Results:**

Our analysis revealed that males had a higher median MOAS score in the first year of clinic when compared with females (median 11, interquartile range [IQR] [4–24] vs. median 3, IQR [1–9]; *p* = 0.03) and a higher median subscore for physical aggression (median 4, IQR [0–12] vs. median 0, IQR [0–8]; *p* = 0.05). The median time from PANS symptom onset to first administration of immunotherapy, which did not include nonsteroidal anti-inflammatory drugs or short bursts of oral steroids, was 6.9 years for females and 3.7 years for males (*p* = 0.20). The two groups did not differ significantly in age of PANS onset, time from onset to clinic entry, other psychiatric symptom measures, or laboratory markers of inflammation.

**Conclusion:**

Among patients with PANS, males exhibit more aggressive behavior when compared with females, which may advance the decision to treat with immunotherapy. Scores that capture a more global level of functioning show that despite there being a higher level of aggression in males, female patients with PANS have similar levels of overall impairment.

## Introduction

Pediatric Acute-onset Neuropsychiatric Syndrome (PANS) is an abrupt-onset neuropsychiatric disease that appears to involve the basal ganglia based on symptoms, striatal autoantibodies, and imaging studies (Giedd et al. 2000; Kumar et al. 2015; Frick et al. 2018; Zheng et al. 2020). It typically follows a relapsing and remitting course (Brown et al. 2017a, 2017b; Hesselmark and Bejerot, 2019). Cardinal symptoms include abrupt onset of significant obsessive–compulsive symptoms, eating restriction, anxiety (especially separation anxiety), emotional lability, impulsivity, behavioral/developmental regression, cognitive symptoms, sensory dysregulation, motor abnormalities, sleep disruption, and enuresis or polyuria (eAppendix in Supplementary Material; Frankovich et al. 2015a, 2015b; Murphy et al. 2015; Toufexis et al. 2015; Gaughan et al. 2016; Calaprice et al. 2017; Santoro et al. 2018; Gamucci et al. 2019; Gromark et al. 2019; Johnson et al. 2019; Silverman et al. 2019). Symptoms may also include irritability with or without aggressive behaviors toward self or others.

PANS is thought to be a postinfectious neuropsychiatric disorder akin to acute rheumatic fever and Sydenham's chorea (Chan and Frankovich 2019). When abrupt-onset and severe obsessive compulsive disorder (OCD) and/or tics is preceded by a streptococcal infection, the syndrome has been termed Pediatric Autoimmune Neuropsychiatric Disorder Associated with Streptococcus (PANDAS) (Swedo et al. 2012; Chang et al. 2015). Two neuroimaging studies in patients with PANDAS suggest neuroinflammation represented by swelling in the basal ganglia during the acute phase of the illness, and microglial activation most prominently in the caudate, putamen, and globus pallidus (basal ganglia) (Giedd et al. 2000; Kumar et al. 2015). A recent MRI study in PANS patients showed greater water diffusivity in all the deep grey matter, most prominently in the thalamus, basal ganglia (caudate, putamen, globus pallidus, etc.) and amygdala of patients suggesting inflammation and/or injury of the brain (Zheng et al. 2020).

Additionally, animal models of PANDAS and Sydenham chorea point to poststreptococcal adaptive immune system activation as a contributor to disease pathogenesis (Hoffman et al. 2004; Yaddanapudi et al. 2010; Brimberg et al. 2012; Cox et al. 2013; Lotan et al. 2014; Cutforth et al. 2016; Dileepan et al. 2016), including a recent study, which showed immunoglobulin G antibodies from children with PANDAS had elevated *ex vivo* binding to striatal cholinergic interneurons, which was associated with reduced signaling of these neurons (Xu et al. 2020).

More broadly, large epidemiological studies have linked preceding streptococcal pharyngitis infections to mental disorders, most prominently OCD and tics in children (Orlovska et al. 2017) and established a familial link between autoimmune diseases and both OCD and Tourette's/chronic tic disorder (Mataix-Cols et al. 2018), which also supports a possible role of immunological factors in etiology. We have also observed a high rate of autoimmunity in family members of our patients with PANS (Chan et al. 2020).

Treatment approaches to PANS/PANDAS are similar to those for Sydenham chorea: clear infections, treat inflammation, and manage psychiatric symptoms based on standard of care (Cooperstock et al. 2017; Frankovich et al. 2017; Thienemann et al. 2017). Initial immunomodulation usually consists of nonsteroidal anti-inflammatory drugs (NSAIDs) and/or brief courses of oral corticosteroids. However, in cases of severe or refractory symptoms, more advanced immunomodulators are often used.

Many diseases affect males and females differently, with the sex of a patient affecting the risk for disease susceptibility and activity by fold differences. For example, systemic lupus erythematosus is nearly ninefold more common in females than males over the lifespan (Izmirly et al. 2017), whereas male patients with systemic lupus erythematosus carry at least threefold higher risk for myocardial infarction than female patients (Vavlukis et al. 2021). In addition, sex differences in immune functioning have been well documented in studies of other inflammatory/autoimmune disorders (Ansar et al. 1985; Oertelt-Prigione 2012). Also, importantly, sex differences in psychiatric conditions have been described and are important to keep in mind (den Braber et al. 2013; Raines et al. 2018).

Thus, we undertook this study to compare males and females with PANS to determine whether sex is associated with differing clinical symptoms. Based on our clinical experience, we hypothesized that males exhibit a higher degree of externalizing behaviors (e.g., aggression), which could translate to higher caregiver burden and influence the decision to treat more aggressively, including influencing the decision to use “early aggressive immunotherapy” (a term used in the autoimmune disease treatment literature), which we defined as the use of: intravenous immunoglobulin (IVIG), intravenous methylprednisolone, prolonged high-dose oral steroids (>1 mg/kg taken for at least 1 month), rituximab, methotrexate, mycophenolate mofetil, and plasmapheresis.

## Methods

### Study location

This study was conducted at Stanford University in affiliation with the Stanford Children's Immune Behavioral Health (IBH) Clinic, a multidisciplinary clinic where patients are followed by psychiatrists, a rheumatologist, an immunologist, a pediatrician or nurse practitioner, and social work psychotherapist and/or psychologist. The Stanford Panel on Human Subjects Institutional Review Board (IRB) approved this study in accordance with the latest version of the Declaration of Helsinki. Informed written consent from parents and adult participants and written assent from competent minor participants were obtained after the nature of the research was fully described and before any data were collected. Data were stored in a secure database.

### Study population

Between September 15, 2012 and March 30, 2019, the IBH Clinic saw 345 unique patients. We excluded patients from the study who refused or withdrew consent (*N* = 8), did not meet PANS or PANDAS criteria (*N* = 103), or lived greater than 90 miles from the clinic at clinic entry (*N* = 29) (Fig. S1 in the Supplementary Appendix). We chose the exclusion criterion for distance because patients who travel from far away may more likely be lost to follow-up. Furthermore, our aim was to evaluate a community cohort to minimize referral center bias. The final study sample included 205 patients. A cohort of healthy controls (*N* = 44) was recruited after the time of this study as a comparison group.

### Sources of data

Data were collected from a clinical research database containing data from clinic-based patient questionnaires completed by caregivers and/or patients. The first author also reviewed electronic medical records to abstract data for additional variables.

### Measures/variables

Demographic and clinical characteristics included age of PANS symptom onset, time from PANS symptom onset to first clinic visit, race, and ethnicity. We also collected the following psychometric scores: (1) the PANS Global Impairment Score, a caregiver-reported measure of disease severity, developed for PANS by Leckman and Murphy that encompasses all PANS symptoms and was recently validated for this patient population (Leibold et al. 2019) and (2) a 24-item Caregiver Burden Inventory (Novak and Guest, 1989), developed to assess caregivers' responses to the demands of caregiving, which was also validated for this patient population (Farmer et al. 2018). Scores of these measures vary with the relapsing–remitting course of PANS (Farmer et al. 2018; Frankovich et al. 2018; Leibold et al. 2019) and to compare patients in their most severe disease states, we utilized the worst scores collected within each patient's first year of follow-up.

On April 15, 2017, the IBH Clinic began administering patient questionnaires in an electronic format, which expanded the paper questionnaire to include more measures. Therefore, only those patients whose first visit was after April 15, 2017 (*N* = 57) were included in the evaluation of these variables. Of these, the measure of primary interest was the Modified Overt Aggression Scale (MOAS), a weighted behavior rating scale scored from 0 (no aggression) to 100 (maximum weighted score) and designed to quantify four types of aggressive behavior as witnessed over a period of 1 week: (1) verbal aggression, (2) property aggression (aggression toward object), (3) autoaggression (aggression toward self), and (4) physical aggression (eAppendix in Supplementary Material).

Investigators reviewed clinical notes to determine whether a patient received aggressive immunotherapy, which we defined as IVIG, intravenous methylprednisolone, prolonged high-dose oral steroids (>1 mg/kg taken for at least 1 month), rituximab, methotrexate, mycophenolate mofetil, and plasmapheresis. For patients who received at least one of these immunotherapies, the date of the first administration was retrospectively identified. Almost all of our patients tried a brief course of “nonaggressive immunotherapy” defined as NSAIDs or brief oral steroid bursts, as these interventions are considered low-risk compared with the alternatives (Brown et al. 2017a, 2017b). For this study, we wanted to differentiate this group of patients from the more severe cases, to whom we restricted the exposure to aggressive immunotherapy along with its concomitant risks.

We also conducted an exploratory analysis of five additional measures (administered with the MOAS in the electronic questionnaire) that were developed for psychiatric and somatic symptoms: (1) the Children's Yale–Brown Obsessive-Compulsive Scale (CY-BOCS), a semistructured instrument for assessing obsessive-compulsive disorder symptom severity in youth (Scahill et al. 1997; Storch et al. 2004; Lewin et al. 2014); (2) the Yale Global Tic Severity Scale (YGTSS), a commonly used index of motor and phonic tic severity in youth (Leckman et al. 1989; Storch et al. 2007; Scahill, 2013); (3) Avoidant/Restrictive Food Intake (ARFI) symptoms, a modified version of the CY-BOCS for thoughts and behaviors regarding eating; (4) the Columbia Impairment Scale, a tool that provides a global measure of functioning (Bird et al. 1993; Attell et al. 2020); and (5) the Children's Global Assessment Scale (CGAS), a clinician-reported measure of the general functioning of youth (Shaffer et al. 1983).

The electronic questionnaire also includes a PANS Symptom Severity Scale, a list of 27 psychiatric, behavioral, and somatic symptoms, for each of which the patient or caregiver provided a severity rating (mild, moderate, severe, or incapacitating). This list was collapsed into eight clinically relevant categories for the exploratory analysis:

1. Disordered eating and drinking
2. Anxiety
3. Mood dysregulation
4. Inhibitory control issues
5. Developmental issues
6. Cognitive issues
7. Somatic symptoms
8. Psychosis

Within each category, the designation of a symptom as severe or incapacitating within the first year of clinic follow-up conferred a positive result; the lack of severe or incapacitating symptoms conferred a negative result for the category.

Lastly, our exploratory analysis evaluated clinical laboratory results from patient's laboratory screening panels at or near their first visits (Chang et al. 2015). Because our aim was to capture levels of laboratory markers during the disease flare state, we collected patient laboratory results within 4 months of the first clinic visit date if the patient was determined by the clinician to be flaring at the first visit. If the patient was remitting by their first visit, we collected laboratory measurements within 4 months of their first flare documented in the clinic. Laboratory values were collected for complement C3, complement C4, vasculitis markers (positive D-dimer and von Willebrand factor), antinuclear antibodies, histone antibodies, antithyroid peroxidase antibodies, and antithyroglobulin antibodies.

### Statistical analysis

We used summary statistics to describe the demographic, clinical, and laboratory characteristics of our study cohort. For continuous variables, we used Student's *t*-tests; for continuous variables that were skewed, we presented medians and used Wilcoxon rank-sum tests. For categorical variables, we conducted chi-square tests or Fisher's exact test if chi-square assumptions were violated. We plotted Kaplan–Meier curves for the time to first immunotherapy by sex, censoring patients at their last follow-up visit, and we compared males with females using the log-rank test. All tests were two tailed.

We also ran two Cox proportional hazards regression models to evaluate the factors that affect time to first immunotherapy. The first model was run on the entire cohort (*N* = 205) and included three potential risk factors, which may hasten time to use of immunotherapy: (1) male sex (primary predictor; externalizing behaviors may make these patients more difficult to manage), (2) age at PANS onset, and (3) at least one comorbid autoimmune disease present at the time of the first clinic visit. The second model was run on the 57 consecutive patients whose first visit was after the implementation of the MOAS survey starting April 15, 2017, and it included the following covariates: (1) MOAS (higher aggression levels is an alarming symptom and may also make patients more difficult to manage), (2) age at PANS onset, and (3) at least one comorbid autoimmune disease diagnosis by the first clinic visit. We included age at PANS onset based on the impression of the clinical team that patients presenting at a younger age are likely to resolve with no or nonaggressive immunomodulation, as may be seen in postinfectious arthritis. This may lead the clinician to hesitate to pursue aggressive immunotherapy in younger children independent of symptom severity.

We included having a comorbid autoimmune disorder as a covariate because these multiple conditions (e.g., rheumatologic and psychiatric symptoms) that present in one patient may likely have a common cause. Since immunotherapy has not been robustly proven to improve long-term outcomes in PANS and in the absence of being able to prove that a patient's psychiatric symptoms are driven by autoimmunity, clinicians may feel more comfortable pursuing immunotherapy if there is a definitive autoimmune disease.

For the PANS Symptom Severity Scale, within each category, proportions of positive results in males and females were compared using chi-square tests or Fisher's exact test whenever appropriate. To assess the likelihood of selection bias, we compared the patients whose first visit was before April 15, 2017 (*N* = 148) with the patients whose first visit was after April 15, 2017 (*N* = 57), and we compared the excluded PANS patients who did not meet the study criterion of distance (*N* = 29) with the entire study cohort (*N* = 205).

Statistical analysis system (SAS) University Edition (SAS Institute, Inc., Cary, NC, USA) was used for statistical analysis.

## Results

### Primary results

Our study cohort comprised 205 consecutive patients with PANS and/or PANDAS—87 females and 118 males. The majority of patients were non-Hispanic white (80%) and the average age in years of PANS symptom onset was slightly lower in females than in males (8.0 ± 3.4 vs. 8.9 ± 3.8, *p* = 0.07) (Table 1). The median time in months from PANS symptom onset to the patient's first clinic visit was not different between males and females (median 8.6, interquartile range [IQR] [2.2–28.1] vs. median 5.6, IQR [2.7–41.7]; *p* = 0.53). The average PANS Global Impairment and Caregiver Burden Inventory scores were both similar between males and females. As a whole, caregiver burden was high; the parents of nearly half of patients had Caregiver Burden Inventory scores at or above 36, a level suggesting a need for respite (Frankovich et al., 2018).

**Table 1.** Demographic and Clinical Characteristics of 205 Consecutive Patients with Pediatric Acute-onset Neuropsychiatric Syndrome

Characteristic	Female (*N* = 87)	Male (*N* = 118)	*p*
	No. (%)	No. (%)	
Age of PANS symptom onset, mean (SD), years	8.0 (3.4)	8.9 (3.8)	0.07
Time from PANS symptom onset to first clinic visit, median [IQR], months	5.6 [2.7–41.7]	8.6 [2.2–28.1]	0.53
Race and ethnicity, *n* (%)			
Non-Hispanic White	72 (82.8)	92 (78.0)	0.40
Other	15 (17.2)	26 (22.0)	
PANS symptoms at clinic presentation, *n* (%)			
OCD	71 (81.6)	94 (79.7)	
Eating restriction	41 (47.1)	42 (35.6)	
Anxiety	71 (81.6)	91 (77.1)	
Emotional lability and/or depression	61 (70.1)	77 (65.3)	
Irritability, aggression, and/or severely oppositional behaviors	54 (62.1)	72 (61.0)	
Behavioral/developmental regression	29 (33.3)	42 (35.6)	
Deterioration in school performance (ADHD-like symptoms, memory deficits, cognitive changes)	43 (49.4)	70 (59.3)	
Sensory dysregulation^a^	35 (40.2)	49 (41.5)	
Motor abnormalities^b^	39 (44.8)	70 (59.3)	
Somatic signs and symptoms (sleep disturbance, involuntary urination, or increased urinary frequency)	62 (71.3)	77 (65.3)	
Homicidal or suicidal ideation/attempts	12 (13.8)	17 (14.4)	
Global Impairment from PANS psychiatric symptoms, mean (SD)^c^	52.1 (25.6)	54.2 (27.5)	0.59
Caregiver Burden Inventory, mean (SD)^c^	37.8 (19.5)	38.8 (21.0)	0.74

^a^ Hyperacusis, photophobia, pain amplification, inability to feel pain, etc.

^b^ Tics, motoric hyperactivity, chorea, etc.

^c^ As these measures vary with the relapsing–remitting course of PANS, the most severe score in the patient's first year of clinic evaluation was used for analysis.

ADHD, attention-deficit/hyperactivity disorder; IQR, interquartile range; OCD, obsessive compulsive disorder; PANS, Pediatric Acute-onset Neuropsychiatric Syndrome; SD, standard deviation.

**FIG. 1.**
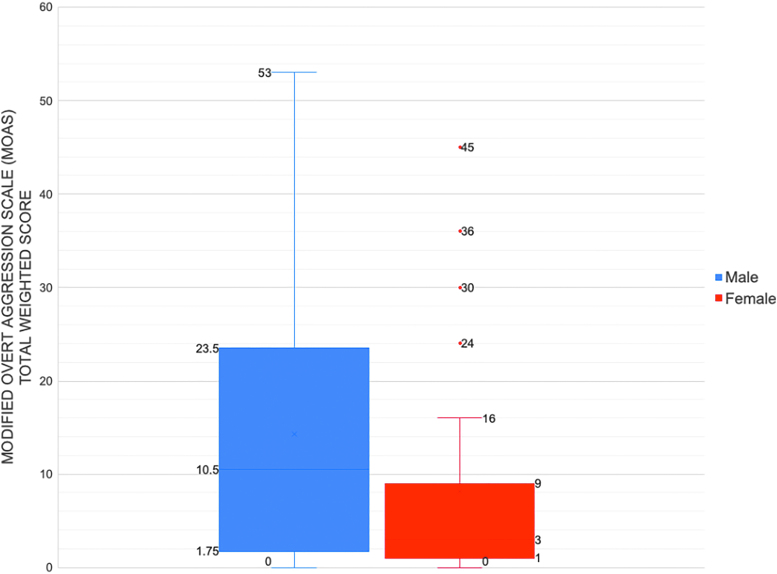
Box-and-whisker plots of the MOAS total score^a^ for 57^b^ consecutive patients in the Stanford PANS study cohort. ^a^We chose the most severe MOAS score for each participant from the initial year of clinic evaluation to compare males to females for two reasons: (1) Since this is a relapsing–remitting disease, patients can present to their first visit either in a relapse or a resolving flare (due to delays in getting into our clinic) and (2) flare intensity varies between flares. ^b^This analysis evaluates MOAS scores of the 57 consecutive patients whose first visit was after the implementation of the MOAS survey in the updated questionnaire starting April 15, 2017. MOAS, Modified Overt Aggression Scale; PANS, Pediatric Acute-onset Neuropsychiatric Syndrome.

We found significant sex differences with regard to the level of aggression, as measured by median total weighted MOAS score and MOAS subscores: males had higher total MOAS scores (median 11, IQR [4–24] vs. median 3, IQR [1–9]; *p* = 0.03). Males also scored notably higher compared with females for the subcategory of physical aggression (median 4, IQR [0–12] vs. median 0, IQR [0–8]; *p* = 0.05) (Table 2 and Fig. 1). For comparison, a cohort of 44 healthy controls (60% male, average age 13.1) was recruited after the time of this study; the average and median MOAS scores among this healthy control cohort were 0 and 0.2, respectively, and the maximum score was 7.

**Table 2.** The Modified Overt Aggression Scale Total Weighted Score and Subscores for 57 Consecutive Patients in the Stanford Pediatric Acute-onset Neuropsychiatric Syndrome Study Cohort

	Female (*N* = 27)		Male (*N* = 30)		
	Median	IQR^b^	Median	IQR^b^	*p*
MOAS total score^c^	3	1–9	10.5	4–24	0.03
Verbal aggression	1	0–1	1	1–3	0.06
Property aggression	0	0–0	0	0–4	0.07
Autoaggression	0	0–3	0	0–3	0.42
Physical aggression	0	0–8	4	0–12	0.05

This analysis evaluates MOAS scores of the 57 consecutive patients whose first visit was after the implementation of the MOAS survey in the updated questionnaire starting April 15, 2017.

^b^ IQR. We chose to report medians and IQR because MOAS scores were highly skewed, with most patients displaying low levels of aggression and a handful of patients having extreme aggression.

^c^ We chose the most severe MOAS score for each participant from the initial year of clinic evaluation to compare males to females for two reasons: (1) Since this is a relapsing–remitting disease, patients often presented to their first PANS clinic visit with a “resolving flare” due to delays in getting into our clinic and (2) flare intensity varies between flares.

IQR, interquartile range; MOAS, Modified Overt Aggression Scale.

**FIG. 2.**
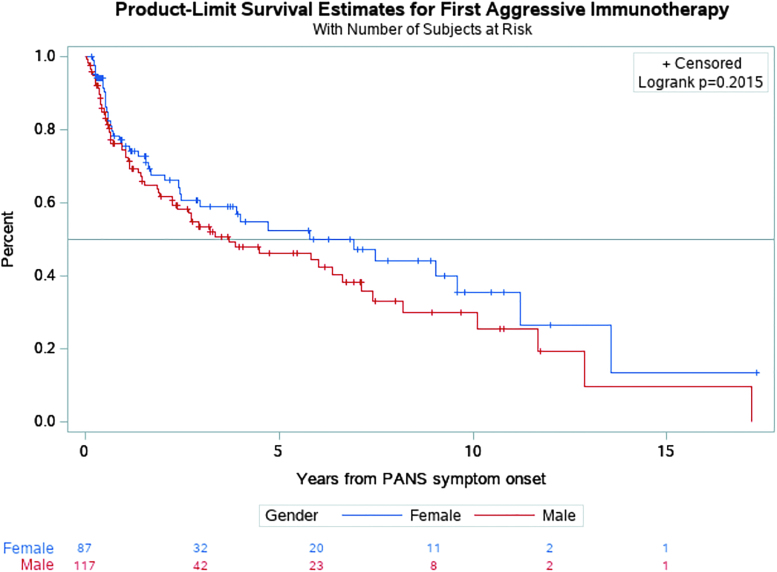
Time from PANS symptom onset to first immunotherapy^a^: The median time to the first administration of aggressive immunotherapy was more than 3 years earlier for males, but this was not significant based on the log-rank test. ^a^Prolonged oral corticosteroids, high-dose IVIG, high-dose intravenous methylprednisolone pulse, rituximab, methotrexate, mycophenolate mofetil, and plasmapheresis. See Table S5 in the Supplementary Appendix for navigational search terms of these therapies. We did not include NSAIDs or brief 3–7-day oral prednisone bursts. IVIG, intravenous immunoglobulin; NSAIDs, nonsteroidal anti-inflammatory drugs; PANS, Pediatric Acute-onset Neuropsychiatric Syndrome.

Kaplan–Meier survival analysis indicates that the median time from PANS symptom onset to first immunotherapy (i.e., IVIG, intravenous methylprednisolone, prolonged high-dose oral steroids [>1 mg/kg taken for at least 1 month], rituximab, methotrexate, mycophenolate mofetil, and plasmapheresis) was 3.7 years for males and 6.9 years for females (*p* = 0.202) (Fig. 2).

In our first Cox proportional hazards model **(***N* = 205, Table 3**)** males had an ∼10.6% lower hazard ratio (HR = 0.894) compared with females; however, this is not significant (*p* = 0.607). On the other hand, with each additional year of PANS onset age, the HR increases by 9.1% (HR = 1.09), a significant change (*p* = 0.003). In other words, patients who are older at the time of their PANS onset are more likely to receive immunotherapy sooner. Diagnosis of a comorbid autoimmune disease did not have a significant effect on time to immunotherapy (*p* = 0.640).

**Table 3.** Using the Cox Proportional Hazards Regression Model to Evaluate Three Potential Predictors of Time to First Immunotherapy: Male Sex (Primary Predictor), Age at Pediatric Acute-Onset Neuropsychiatric Syndrome Onset, and Comorbid Autoimmune Disease Diagnosed by the First Clinic Visit

Risk factor	*N* = 205		
	No. (%)	HR (95% CI)^a^	*p*
Male sex	118 (57.6%)	0.894 (0.583–1.371)	0.6068
Age at PANS onset, mean (SD), years	8.5 (3.6)	1.091 (1.031–1.154)	0.0025
Comorbid autoimmune disease^b^	16 (7.8%)	0.829 (0.377–1.823)	0.6403

Although males have an ∼10.6% (HR = 0.894) decrease in the HR compared with females, this decrease is not significant. On the other hand, with each additional year of PANS onset age, the HR increases by 9.1% (HR = 1.091), a significant change. In other words, patients who are older at the time of their PANS onset are more likely to receive immunotherapy sooner. Diagnosis of a comorbid autoimmune disease did not have a significant effect on risk of immunotherapy.

^a^ The analysis was adjusted for race and ethnicity.

^b^ Diagnosed by the first clinic visit.

HR, hazard ratio; PANS, Pediatric Acute-onset Neuropsychiatric Syndrome; SD, standard deviation.

In our second Cox proportional hazards model (*N* = 57; Table 4), a one-point increase in MOAS score is significantly associated with a 3.8% increase (HR = 1.038, *p* = 0.013) in the expected risk of receiving immunotherapy. As in the first model, older age of onset of PANS is associated with increased risk of receiving immunotherapy (*p* = 0.004), and diagnosis of a comorbid autoimmune disease did not have a significant effect (0.322).

**Table 4.** Using the Cox proportional Hazards Regression Model to Evaluate the Association Between the Time to First Immunotherapy and Three Suspected Risk Factors: Modified Overt Aggression Scale (Primary Predictor), Age at Pediatric Acute-Onset Neuropsychiatric Syndrome Onset, and Comorbid Autoimmune Disease Diagnosed by the First Clinic Visit

Risk factor	*N* = 57^a^		
	No. (%)	HR (95% CI)^b^	*p*
MOAS, median [IQR]	9 [1–16]	1.038 (1.008–1.068)	0.0131
Age at PANS onset, mean (SD), years	8.2 (3.3)	1.214 (1.062–1.387)	0.0044
Comorbid autoimmune disease^c^	3 (5.3%)	1.944 (0.522–7.240)	0.3215

A one-point increase in MOAS score is significantly associated with a 3.8% increase (HR = 1.038) in the expected hazard of receiving immunotherapy. As in the previous model, an older age of onset of PANS is associated with increased risk of receiving immunotherapy, and diagnosis of a comorbid autoimmune disease did not have a significant effect on risk of immunotherapy.

^a^ This analysis evaluates MOAS scores of the 57 consecutive patients whose first visit was after the implementation of the MOAS survey in the updated questionnaire starting April 15, 2017.

^b^ The analysis was adjusted for race and ethnicity.

^c^ Diagnosed by the first clinic visit.

HR, hazard ratio; IQR, interquartile range; MOAS, Modified Overt Aggression Scale; PANS, Pediatric Acute-onset Neuropsychiatric Syndrome; SD, standard deviation.

### Results of exploratory analyses

We did not find significant sex differences in other clinical measures at presentation; in the first year of clinic follow-up, males and females had similar results for the CY-BOCS, YGTSS, ARFI, Columbia Impairment, and CGAS (Table S1 in the Supplementary Appendix).

Of the eight PANS symptom categories, a difference across sex was found only in the inhibitory control category, which includes hyperactivity, impulsivity, oppositionality, and trouble paying attention. Seventeen males (53%) compared with seven females (25%; *p* = 0.03) received scores of severe or incapacitating dysfunction in this category **(**Table S2 in the Supplementary Appendix**)**.

Neither complement levels (C3, C4), serum vasculitis markers (von Willebrand factor and D-dimer), nor autoantibodies differed significantly between males and females. A higher proportion of males compared with females had low complement levels (37% vs. 30%) and vasculitis markers (15% vs. 10%), whereas a higher proportion of females had positive autoantibodies compared with males (30% vs. 25%) (Table S3 in the Supplementary Appendix).

### Results of sensitivity analysis

Our sensitivity analysis comparing the patients whose first visit was before April 15, 2017 (*N* = 148) (i.e., no MOAS survey administered) to the patients whose first visit was after April 15, 2017 (*N* = 57) (i.e., MOAS survey administered at every clinic visit) showed that the groups were similar with regard to age of PANS onset, race, and PANS Global Impairment Score (Table S4 in the Supplementary Appendix). The post-April 2017 cohort had a lower median time from PANS onset to first clinic visit (*p* = 0.006), which could be a result of the fact that clinicians were able to see patients sooner as our clinic obtained more resources (dedicated patient care coordinator and additional clinicians). The post-April 2017 cohort also had a lower average Caregiver Burden Inventory (*p* = 0.04), which may be a result of less delay in receiving care.

Our comparison of the excluded PANS patients who lived >90 miles of our clinic (*N* = 29) to those who were included (living within 90 miles) (*N* = 205) showed that the two groups were similar, except that the excluded cohort had a higher median time from PANS onset to first clinic visit (*p* = 0.048).

## Discussion

The largest studies of patients with PANS report a male predominance ranging from 56% to 77% (Swedo et al. 1998; Frankovich et al. 2015a; Calaprice et al. 2017; Gromark et al. 2019), but none of these studies showed statistical significance. Our consecutive patient study is the largest to date and found a similarly high but also insignificant male preponderance of 58%. In 2015, due to lack of security to keep staff and families safe, our clinic began to screen out patients with known risk for violence to clinical staff. However, we continued to accept patients who have minimal known risk of injury or harm, which may explain the high proportion of aggressive patients in our clinic. This policy was potentially biased toward a higher case acceptance of females, with less-reported physical aggression and may help explain why we did not observe a higher male/female ratio seen in other observational studies.

Our primary analysis found that males did exhibit more aggressive behavior than females, based on their higher MOAS scores (Table 2), supporting our primary hypothesis that males have exhibited more externalizing behaviors, such as aggression. Although the physical aggression measured by the MOAS was significantly higher in males, caregivers of males and females reported similar levels of caregiver burden. Impairment levels, as estimated by clinicians by the CGAS, and parents as estimated by the PANS Global Impairment score and Columbia Impairment Scale, were similar in males and females as well. These findings indicate that the impact of PANS on parenting children of the two sexes may not be different enough to affect the burden of caregiving and that female patients have symptoms that are challenging to manage despite having lower aggression levels.

Along with management of infections and psychiatric support, immunotherapy constitutes an important part of PANS clinical care (Swedo et al. 2017). Our initial approach to immunotherapy utilizes “nonaggressive” NSAIDs and/or brief oral corticosteroid bursts, particularly for younger patients, those with lower severity and higher functioning. However, we do employ aggressive immunotherapy in select patients based on a multifactorial decision process. As these immunotherapies may pose significant risks, it is important to understand the factors that affect treatment selection, and we had hypothesized that a child with aggressive behavior would influence families' and clinicians' sense of urgency for aggressive immunotherapy.

After accounting for age at PANS onset and diagnosis of a comorbid autoimmune disease at the first visit, we found that the association between MOAS score and aggressive immunotherapy is more significant than the sex effect, which suggests that physical and verbal aggression may explain the earlier treatment of males with aggressive immunotherapy. When a patient is verbally or physically aggressive toward other people, failure to control the underlying disease may have more dire consequences, especially when their symptoms are refractory to standard psychiatric treatments. Similarly, in the German health system, patients with externalizing symptoms (conduct disorders, including oppositional defiant disorders) were more prone to receive medication and/or psychotherapy than the average child diagnosed with attention-deficit/hyperactivity disorder (Scholle et al. 2020). However, in all inflammatory diseases, the decision to treat with immunotherapy is multifactorial and complex, and it takes into account both the trajectory and the severity of illness.

We did find that the older age of onset of PANS was also associated with an increased likelihood of receiving aggressive immunotherapy, but the reasons are unclear. In our clinical practice, we generally limit immunomodulation (IVIG, IV steroids, etc.) to the more severe patients who have (1) a high burden of autoimmune markers, (with or without a formal diagnosis of a comorbid autoimmune disease), (2) more chronic disease at presentation, and (3) a history of poor response to psychiatric interventions. However, these explanations need to be further explored.

In this study cohort, the median time from PANS symptom onset to first immunotherapy was remarkably long for both groups (3.7 years for males and 6.9 years for females), which likely reflects a number of factors, including delay in diagnosis and access to our clinic. Additionally, for most of our patients who experience their first episode at a young age and have a relapsing–remitting course of disease—with flare duration typically lasting 3 months—exposing children to powerful immunomodulatory agents at an early stage was not justified. In this early-onset population, the relapsing–remitting disease course is similar to childhood asthma, and we approach exacerbations similarly with milder treatments, including NSAIDs and prednisone bursts (Brown et al. 2017a, 2017b).

The main strengths of this study are the protocol-driven approach of data collection and the multidisciplinary nature of our clinic. However, both parent-rated and physician-rated clinical scores are subjective and may have inherent biases. Our cohort is biased toward youth with less physical aggression against others as a result of institutional policy, as discussed above, such that our resulting cohort is not representative of the full spectrum of behaviors in patients with PANS. At the same time, the evidence of increased general aggression in males in the context of excluding violent patients may give weight to this finding.

## Conclusion

Among youth meeting criteria for PANS, males exhibit a higher level of aggressive behavior when compared with females, which may advance the decision to treat with immunotherapy. Despite this, scores that capture a more global level of functioning show that males and females with PANS are equally impaired. Longitudinal follow-up of these patients in our clinic will allow for more sophisticated analyses and a deeper understanding of this patient group with regard to multifactorial decisions such as treatment.

## Clinical Significance

Female patients with PANS have similar levels of impairment as their male counterparts, who on average display a higher level of verbal and physical aggression. In treating patients with PANS, clinicians should be aware that these externalizing symptoms may influence treatment decisions and risk–benefit discussions between the team and family. However, the decision to employ aggressive immunotherapy should be based on a comprehensive evaluation of the clinical situation, including efficacy of psychiatric interventions, biomarkers suggesting comorbid autoimmunity, and all available measures of impairment.

## Supplementary Material

Supplemental data

Supplemental data

Supplemental data

Supplemental data

Supplemental data

Supplemental data

Supplemental data
