# Supplementary material for: Sex and Aggression Characteristics in a Cohort of Patients with Pediatric Acute-Onset Neuropsychiatric Syndrome
Source: J Child Adolesc Psychopharmacol. 2022 Oct 17;32(8):444–52. doi: 10.1089/cap.2021.0084 (PMC9603278; doi:10.1089/cap.2021.0084)
Supplement: Supplemental data [file Suppl_Appendix.pdf]

Supplemental Appendix

eAppendix. Criteria for Diagnosis of PANS

1. Abrupt, dramatic onset of obsessive-compulsive disorder or severely restricted food intake
2. Concurrent presence of additional neuropsychiatric symptoms, with similarly severe and acute onset, from at least two of the following seven categories:
    a. Anxiety
    b. Emotional lability and/or depression
    c. Irritability, aggression and/or severely oppositional behaviors
    d. Behavioral (developmental) regression
    e. Deterioration in school performance
    f. Sensory or motor abnormalities
    g. Somatic signs and symptoms, including sleep disturbances, enuresis or urinary frequency
3. Symptoms are not better explained by a known neurologic or medical disorder, such as Sydenham chorea, systemic lupus erythematosus, Tourette disorder or others.

Figure 1: Participant flow diagram with exclusion criteria.

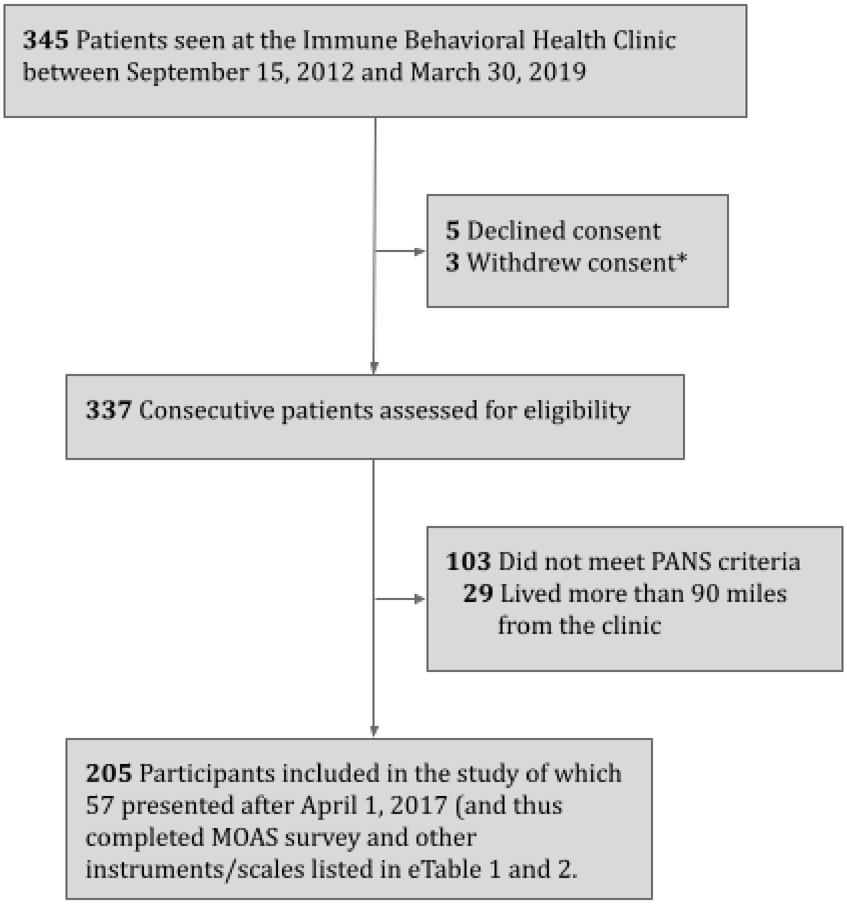

*One patient was withdrawn because their consent form was filled out incorrectly.

Table 1: Exploratory analysis of other psychiatric symptom scores of consecutive patients whose first visit was after April 15, 2017 (N=57)

	**Female (N=27)**	**Male (N=30)**	**p-value**
	**Mean (SD)**	**Mean (SD)**	
CY-BOCS[1]	20.6 (8.7)	20.4 (9.0)	0.92
Avoidant/Restrictive Food Intake	17.5 (9.4)	13.8 (10.6)	0.27
Yale Global Tic Severity Scale, *median [IQR]*	14 [0-35]	27 [6-45]	0.08[2]
Columbia Impairment	15.6 (9.2)	18.4 (11.7)	0.35
C-GAS[3]	56.3 (19.5)	50.5 (15.6)	0.22

[1]Children's Yale-Brown Obsessive Compulsive Scale, a semi-structured instrument for assessing obsessive compulsive disorder symptom severity in youth (Scahill et al., 1997; Storch et al. 2004; Lewin et al., 2013)

[2]Wilcoxon rank-sum two-tailed Z

[3]Children's Global Assessment Scale, a clinician-reported measure of the general functioning of youth (Shaffer et al., 1983)

Table 2. Severe or incapacitating symptoms within the first year of clinic evaluation (N=57)

	**Female (N=27)**	**Male (N=30)**	**p-value**
	N (%)	N (%)	
Disordered eating and drinking<br>Food refusal/avoidance<br>Urge to overeat<br>Fluid refusal/avoidance	5 (18)	5 (16)	0.82
Anxiety<br>Separation anxiety<br>Other anxiety/fear/phobias/panic attacks	14 (50)	11 (34)	0.22
Mood dysregulation<br>Mood swings/moodiness<br>Emotional lability<br>Suicidal ideation/behavior<br>Depression/sadness<br>Irritability	14 (50)	15 (47)	0.81
Inhibitory control issues<br>Oppositional behaviors<br>Hyperactivity or impulsivity<br>Trouble paying attention	7 (25)	17 (53)	0.03
Developmental issues<br>Baby talk<br>Other behavioral/developmental regression	3 (10)	7 (22)	0.25
Cognitive issues:<br>Worsening of school performance<br>Worsening of handwriting/copying/art<br>Cognitive symptoms (e.g., difficulty thinking, memory problems)	8 (29)	11 (34)	0.63
Somatic symptoms<br>Pain (e.g., headaches, abdominal pain)<br>Sleep disturbance<br>Enuresis<br>Urinary frequency<br>Sensory amplification	8 (29)	14 (44)	0.22
Psychosis:<br>Hallucinations<br>Delusions or paranoid thoughts	2 (7)	6 (19)	0.19

Table 3. Clinical laboratory results for labs completed within four months of clinic entry

	Female	Male	p-value
**Characteristic**	N (%)	N (%)	
Low complement (C3 or C4)	13/44 (30%)	19/52 (37%)	0.43
Any autoantibodies[a]	21/71 (30%)	23/92 (25%)	0.51
Vasculitis markers[b]	4/39 (10%)	7/48 (15%)	0.55

[a]Measured by the clinical team and includes Antinuclear antibodies (ANA), histone antibodies, and thyroid antibodies (thyroperoxidase antibody and anti-thyroglobulin antibody)
[b]Vasculitis markers include Von Willebrand factor (vWF) and D-dimer

Table 4. Sensitivity analysis comparing 1) patients whose first visit was before April 15, 2017 (N=148) to those whose first visit was after April 15, 2017 (N=57) and 2) the study cohort (N=205) to patients meeting PANS criteria who were excluded because they lived more than 90 miles from the clinic (N=29).

	**pre-April 2017 cohort (N=148)**	**post-April 2017 cohort (N=57)**	**p-value**	**Study cohort (N=205)**	**>90 mi cohort (N=29)**	**p-value**
**Characteristic**		N (%)		N (%)	N (%)	
Age of PANS symptom onset, *mean (SD), years*	8.6 (3.8)	8.2 (3.3)	0.45	8.5 (3.6)	7.8 (3.6)	0.29
Time from PANS symptom onset to first clinic visit, *median [IQR], months*	11.3 [3.0-40.3]	2.9 [1.9-18.8]	0.0057	6.9 [2.5-35.0]	19.9 [7.0-49.6]	0.0484
Race and ethnicity, N (%)						
Non-Hispanic White	122 (82.4%)	42 (73.7%)	0.16	164 (80.0%)	26 (89.7%)	0.21
Other	26 (17.6%)	15 (26.3%)		41 (20.0%)	3 (10.3%)	
Global Impairment from PANS psychiatric symptoms, mean (SD)	53.9 (27.5)	51.9 (24.7)	0.64	53.3 (26.6)	52.3 (27.5)	0.85
Caregiver Burden Inventory, mean (SD)	40.5 (20.8)	33.6 (18.4)	0.04	38.3 (20.3)	40.9 (21.4)	0.61

Table 5. Search terms used to find aggressive immunotherapy in the electronic health record search function.

Therapy	Search Terms
Prolonged oral steroids >1mg/kg for >1 month	steroids, pred (for prednisone and prednisolone), methylpred, decadron
Intravenous immunoglobulin	IVIG
Methylprednisolone	Solumedrol, methylpred
Rituximab	ritux
Methotrexate	Methotrexate, mtx
Mycophenolate mofetil	Cellcept, Myfortic, Mycofenolate mofetil, MMF
Plasmapheresis	pheresis, pex

THE MODIFIED OVERT AGGRESSION SCALE (MOAS)*

Patient ____

Rater ______________________________ Date ____________

INSTRUCTIONS

Rate the patient's aggressive behavior over the past week. Select as many items as are appropriate.
Refer to the pocket guide for the full measure.

SCORING

1. Add items in each category
2. In scoring summary, multiply sum by weight and add weighted sums for total weighted score. Use this score to track changes in level of aggression over time.

Verbal aggression

_____ **0** No verbal Aggression
_____ **1** Shouts angrily, curses mildly, or makes personal insults
_____ **2** Curses viciously, is severely insulting, has temper outbursts
_____ **3** Impulsively threatens violence toward others or self
_____ **4** Threatens violence toward others or self repeatedly or deliberately
_____ **SUM VERBAL AGGRESSION SCORE**

Aggression against Property

_____ **0** No aggression against property
_____ **1** Slams door, rips clothing, urinates on floor
_____ **2** Throws objects down, kicks furniture, defaces walls
_____ **3** Breaks objects, smashes windows
_____ **4** Sets fires, throws objects dangerously
_____ **SUM PROPERTY AGGRESSION SCORE**

Autoaggression

_____ **0** No autoaggression
_____ **1** Picks or scratches skin, pulls hair out, hits self (without injury)
_____ **2** Bangs head, hits fists into walls, throws self onto floor
_____ **3** Inflicts minor cuts, bruises, burns, or welts on self
_____ **4** Inflicts major injury on self or makes a suicide attempt
_____ **SUM AUTOAGGRESSION SCORE**

Physical Aggression

_____ **0** No physical aggression
_____ **1** Makes menacing gestures, swings at people, grabs at clothing
_____ **2** Strikes, pushes, scratches, pulls hair of others (without injury)
_____ **3** Attacks others, causing mild injury (bruises, sprain, welts, etc.)
_____ **4** Attacks others, causing serious injury
_____ **SUM PHYSICAL AGGRESSION SCORE**

CATEGORY	SUM SCORE	WEIGHTS	WEIGHTED SUM
Verbal Aggression		x 1	
Aggression against Property		x 2	
Autoaggression		x 3	
Physical Aggression		x 4	
**Total Weighted Score**			

*Modified from Kay SR, Wolkenfelf F, Murrill LM (1988), Profiles of aggression among psychiatric patients: I. nature and prevalence. *Journal of Nervous and Mental Disease 176*:539-546

The recommendations in this publication do not indicate an exclusive course of treatment or serve as a standard of medical care. Variations, taking into account individual circumstances, may be appropriate. Original document included as part of *Addressing Mental Health Concerns in Primary Care: A Clinician's Toolkit.* Copyright © 2010 American Academy of Pediatrics. All Rights Reserved. The American Academy of Pediatrics does not review or endorse any modifications made to this document and in no event shall the AAP be liable for any such changes.
