# Supplementary material for: Sex and Aggression Characteristics in a Cohort of Patients with Pediatric Acute-Onset Neuropsychiatric Syndrome
Source: J Child Adolesc Psychopharmacol. 2022 Oct 17;32(8):444–52. doi: 10.1089/cap.2021.0084 (PMC9603278; doi:10.1089/cap.2021.0084)
Supplement: Supplemental data [file Suppl_TableS5.pdf]

Table 5. Search terms used to find aggressive immunotherapy in the electronic health record search function.

Therapy	Search Terms
Prolonged oral steroids >1mg/kg for >1 month	steroids, pred (for prednisone and prednisolone), methylpred, decadron
Intravenous immunoglobulin	IVIG
Methylprednisolone	Solumedrol, methylpred
Rituximab	ritux
Methotrexate	Methotrexate, mtx
Mycophenolate mofetil	Cellcept, Myfortic, Mycofenolate mofetil, MMF
Plasmapheresis	pheresis, pex
