# Supplementary material for: Sex and Aggression Characteristics in a Cohort of Patients with Pediatric Acute-Onset Neuropsychiatric Syndrome
Source: J Child Adolesc Psychopharmacol. 2022 Oct 17;32(8):444–52. doi: 10.1089/cap.2021.0084 (PMC9603278; doi:10.1089/cap.2021.0084)
Supplement: Supplemental data [file Suppl_TableS1.pdf]

Table 1: Exploratory analysis of other psychiatric symptom scores of consecutive patients whose first visit was after April 15, 2017 (N=57)

	**Female (N=27)**	**Male (N=30)**	**p-value**
	**Mean (SD)**	**Mean (SD)**	
CY-BOCS[1]	20.6 (8.7)	20.4 (9.0)	0.92
Avoidant/Restrictive Food Intake	17.5 (9.4)	13.8 (10.6)	0.27
Yale Global Tic Severity Scale, *median [IQR]*	14 [0-35]	27 [6-45]	0.08[2]
Columbia Impairment	15.6 (9.2)	18.4 (11.7)	0.35
C-GAS[3]	56.3 (19.5)	50.5 (15.6)	0.22

[1]Children's Yale-Brown Obsessive Compulsive Scale, a semi-structured instrument for assessing obsessive compulsive disorder symptom severity in youth (Scahill et al., 1997; Storch et al. 2004; Lewin et al., 2013)

[2]Wilcoxon rank-sum two-tailed Z

[3]Children's Global Assessment Scale, a clinician-reported measure of the general functioning of youth (Shaffer et al., 1983)
