# Supplementary material for: Sex and Aggression Characteristics in a Cohort of Patients with Pediatric Acute-Onset Neuropsychiatric Syndrome
Source: J Child Adolesc Psychopharmacol. 2022 Oct 17;32(8):444–52. doi: 10.1089/cap.2021.0084 (PMC9603278; doi:10.1089/cap.2021.0084)
Supplement: Supplemental data [file Suppl_TableS2.pdf]

Table 2. Severe or incapacitating symptoms within the first year of clinic evaluation (N=57)

	Female (N=27)	Male (N=30)	p-value
	N (%)	N (%)	
Disordered eating and drinking<br>Food refusal/avoidance<br>Urge to overeat<br>Fluid refusal/avoidance	5 (18)	5 (16)	0.82
Anxiety<br>Separation anxiety<br>Other anxiety/fear/phobias/panic attacks	14 (50)	11 (34)	0.22
Mood dysregulation<br>Mood swings/moodiness<br>Emotional lability<br>Suicidal ideation/behavior<br>Depression/sadness<br>Irritability	14 (50)	15 (47)	0.81
Inhibitory control issues<br>Oppositional behaviors<br>Hyperactivity or impulsivity<br>Trouble paying attention	7 (25)	17 (53)	0.03
Developmental issues<br>Baby talk<br>Other behavioral/developmental regression	3 (10)	7 (22)	0.25
Cognitive issues:<br>Worsening of school performance<br>Worsening of handwriting/copying/art<br>Cognitive symptoms (e.g., difficulty thinking, memory problems)	8 (29)	11 (34)	0.63
Somatic symptoms<br>Pain (e.g., headaches, abdominal pain)<br>Sleep disturbance<br>Enuresis<br>Urinary frequency<br>Sensory amplification	8 (29)	14 (44)	0.22
Psychosis:<br>Hallucinations<br>Delusions or paranoid thoughts	2 (7)	6 (19)	0.19
