# Supplementary material for: Sex and Aggression Characteristics in a Cohort of Patients with Pediatric Acute-Onset Neuropsychiatric Syndrome
Source: J Child Adolesc Psychopharmacol. 2022 Oct 17;32(8):444–52. doi: 10.1089/cap.2021.0084 (PMC9603278; doi:10.1089/cap.2021.0084)
Supplement: Supplemental data [file Suppl_TableS3.pdf]

Table 3. Clinical laboratory results for labs completed within four months of clinic entry

	**Female**	**Male**	**p-value**
**Characteristic**	N (%)	N (%)	
Low complement (C3 or C4)	13/44 (30%)	19/52 (37%)	0.43
Any autoantibodies[a]	21/71 (30%)	23/92 (25%)	0.51
Vasculitis markers[b]	4/39 (10%)	7/48 (15%)	0.55

[a]Measured by the clinical team and includes Antinuclear antibodies (ANA), histone antibodies, and thyroid antibodies (thyroperoxidase antibody and anti-thyroglobulin antibody)

[b]Vasculitis markers include Von Willebrand factor (vWF) and D-dimer
