# Supplementary material for: Sex and Aggression Characteristics in a Cohort of Patients with Pediatric Acute-Onset Neuropsychiatric Syndrome
Source: J Child Adolesc Psychopharmacol. 2022 Oct 17;32(8):444–52. doi: 10.1089/cap.2021.0084 (PMC9603278; doi:10.1089/cap.2021.0084)
Supplement: Supplemental data [file Suppl_TableS4.pdf]

Table 4. Sensitivity analysis comparing 1) patients whose first visit was before April 15, 2017 (N=148) to those whose first visit was after April 15, 2017 (N=57) and 2) the study cohort (N=205) to patients meeting PANS criteria who were excluded because they lived more than 90 miles from the clinic (N=29).

	**pre-April 2017 cohort (N=148)**	**post-April 2017 cohort (N=57)**	**p-value**	**Study cohort (N=205)**	**>90 mi cohort (N=29)**	**p-value**
**Characteristic**		N (%)		N (%)	N (%)	
Age of PANS symptom onset, *mean (SD), years*	8.6 (3.8)	8.2 (3.3)	0.45	8.5 (3.6)	7.8 (3.6)	0.29
Time from PANS symptom onset to first clinic visit, *median [IQR], months*	11.3 [3.0-40.3]	2.9 [1.9-18.8]	0.0057	6.9 [2.5-35.0]	19.9 [7.0-49.6]	0.0484
Race and ethnicity, N (%)						
Non-Hispanic White	122 (82.4%)	42 (73.7%)	0.16	164 (80.0%)	26 (89.7%)	0.21
Other	26 (17.6%)	15 (26.3%)		41 (20.0%)	3 (10.3%)	
Global Impairment from PANS psychiatric symptoms, mean (SD)	53.9 (27.5)	51.9 (24.7)	0.64	53.3 (26.6)	52.3 (27.5)	0.85
Caregiver Burden Inventory, mean (SD)	40.5 (20.8)	33.6 (18.4)	0.04	38.3 (20.3)	40.9 (21.4)	0.61
